# Adaptation and Validation of the Polish Version of the 10-Item Mobile Phone Problematic Use Scale

**DOI:** 10.3389/fpsyt.2020.00427

**Published:** 2020-06-03

**Authors:** Anna Mach, Marta Demkow-Jania, Anna Klimkiewicz, Andrzej Jakubczyk, Małgorzata Abramowska, Anna Kuciak, Piotr Serafin, Jan Szczypiński, Marcin Wojnar

**Affiliations:** ^1^Department of Psychiatry, Medical University of Warsaw, Warsaw, Poland; ^2^Nowowiejski Psychiatric Hospital, Warsaw, Poland; ^3^Polish Society for Prevention of Drug Abuse, Warsaw, Poland; ^4^Laboratory of Brain Imaging, Nencki Institute of Experimental Biology, Polish Academy of Sciences, Warsaw, Poland; ^5^Department of Psychiatry, University of Michigan, Ann Arbor, MI, United States

**Keywords:** mobile phone use, problematic mobile phone use, MPPUS-10, technological addictions, validation, psychometric properties

## Abstract

Pathological use of smartphones may be the biggest non-drug addiction of the 21st century. Therefore, rapid screening tools designed for easy identification of people with problematic mobile phone use are needed. The main aim of the present study was to validate a short version of the Mobile Phone Problematic Use Scale (MPPUS-10) in the Polish population. The study comprised 640 university students aged 18–38 years. We used a self-report questionnaire that included questions regarding socio-demographic variables and Polish versions of the *Mobile Phone Problem Use Scale (MPPUS-10), Mobile Phone Addiction Assessment Questionnaire (MPAAQ* in Polish *KBUTK)*, and *Internet Addiction Test (IAT) by Kimberly Young*. The analysis showed high reliability for the final Polish version of MPPUS-10 (Cronbach’s α = 0.78) and confirmed a significant correlation between the MPPUS-10 and the MPAAQ, which was previously used in Poland (rho = 0.56; p < 0.001). Due to the poor correlation of item number 10 with other items, we suggest dropping this item and using the nine-item Polish version. Our result also confirmed significant correlation between the MPPUS-10 and the IAT (rho = 0.54; p < 0.001). The contribution of the study is the Polish validation and adaptation of the MPPUS-10 scale with confirmed psychometric values. It provides a quick and convenient screening tool to assess problematic mobile phone use. Our results also indicate the need for a revision of available diagnostic tools in Poland.

## Introduction

The 21st century is characterized by major development of modern technologies and a growing number of users of those technologies. The use of new technologies such as the Internet, mobile phones, or smartphones undoubtedly has a wide range of beneficial effects. However, overuse can be associated with harmful and problematic behaviors (1–3). Numerous studies have demonstrated that overuse of modern technologies may have a negative impact on physical health, including inducing headache (4), concentration difficulties (2), pain, fatigue (5), reduction in the amount of physical activity (1), and indirect injuries such as accidents affecting pedestrians or drivers (3, 6). Moreover, excessive mobile phone use has been linked to sleep disturbances, symptoms of depression (2), anxiety (7), worse academic performance (8, 9), or dissatisfaction with life (10).

Despite the evidence for negative consequences associated with the use of modern technologies, research studies have not provided sufficient evidence to include smartphone addiction as a distinct disorder in the current classifications ICD-10 (International Classification of Diseases) (11) and the most recent version of the Diagnostic and Statistical Manual for Mental Disorders (DSM-5) (12). However, taking into consideration the occurrence of symptoms similar to those observed in substance use or gambling (tolerance, impaired control, withdrawal symptoms, intense desire, social problems), some researchers suggest that technological addiction may be treated as a behavioral addiction (13–15). Lack of a universal definition of the disorder is associated with the inconsistency of the terminology used to describe problematic mobile phone use. Several terms have been used to describe the behavior of continuous use of a mobile phone (or smartphone) in spite of negative outcomes: problematic mobile phone use (PMPU), dysfunctional use of the mobile phone, mobile phone dependence syndrome, mobile phone addiction, compulsive mobile phone use, or phonoholism (15–20).

The diagnostic tool for assessing problematic mobile phone use, considered to be a benchmark scale, is the Mobile Phone Problem Use Scale (MPPUS-27) (16). The original version and its modifications have been used in research on the problematic use of telephones in Great Britain (21), Spain (22, 23), Greece (24), Sweden (18), Switzerland (25), Germany (26), Iran (27, 28), Japan (29), and Turkey (30, 31).

The MPPUS-27 (27 items) was originally created for an adult population, and it was then adopted and validated for a large range of ages (23, 26, 28, 29). Foerster and colleagues have introduced a short version (MPPUS-10), which is highly representative of the original MPPUS-27 (18). It consists of only 10 short, easy to understand items and has been shown to have strong discriminatory power.

In Poland, there is a lack of a validated screening tool that would enable easy and rapid identification of people with problematic use of mobile phones. Therefore, the main objective of this study was the validation and adaptation of the Polish version of the MPPUS-10. We chose to conduct our research on a group of students and young adults because previous research has confirmed that the problematic use of mobile phones is mostly relevant to this age group (22, 32–34). Also, the short, 10-item version of MPPUS has previously been used to study this age group (18–33 years) (35). Undoubtedly, this is the first generation that has so much access to modern technologies (36). Providing a quick, reliable tool to identify this disorder may contribute to early intervention.

## Material and methods

### Translation and Adaptation

The MPPUS-10 was translated into Polish in two phases according to WHO standards. First, the scale was translated from English to Polish. Translation was performed by experienced medical doctors with fluent English. The next step was back-translation from Polish to English by an independent translator. We then asked the author of the original MPPUS-10 for a review of our translation. After it was approved by the author, we checked the readability of the Polish version of the MPPUS-10 through a pilot study among medical students (n = 40).

### Participants

The study was conducted according to the recommendations of the Declaration of Helsinki in 1964 and received approval from the Bioethics Committee at the Medical University of Warsaw. All respondents were informed about the course and the aims of the study and gave their informed consent to participate. Participation was voluntary and anonymous. Collected data were confidential. The study involved adult volunteers aged 18–40. The exclusion criteria for the study included: age below 18 or above 40 years, lack of informed consent, inability to complete the survey or to understand the purpose of the study.

Warsaw Medical University students (US; n = 640) were asked to fill out the experimental survey during their medical courses. All participants completed the self-report questionnaires in writing format by selecting their preferred answers. The assessment took place during one meeting without a time limit. Questionnaires were then collected and analyzed by specialists.

For each analysis, participants with missing data were excluded. The reliability and factor analyses of the MPPUS-10 was conducted on 629 US (Age, M = 20.7; SD = 1.95). The validity analysis of the MPPUS-10 was conducted on 530 US (Age, M = 20.7; SD = 1.94), due to missing data in other questionnaires. The demographic data are presented in **Table 1**.

**Table 1:** Demographic and questionnaire data for university students (n=530).

Gender	n (%)	
female	325 (61.3%)	
male	205 (38.7%)	
	
**Where did you live before your studies?**		
village	95 (18%)	
town, population up to 5 thousand	16 (3%)	
town, population from 5 to 20 thousand	57 (10.8%)	
city, population from 20 to 100 thousand	114 (21.5%)	
city, population greater than 100 thousand	248 (46.7%)	
	
**How would you describe your material status?**		
bad	3 (0.6%)	
satisfactory	76 (14.3%)	
good	270 (50.9%)	
very good	181 (34.2%)	
	
	**Median**	**IQR**
**Age**	20	3
**IAT**	30	10
**MPPUS-10**	35	21.75
**MPAAQ**	31	17

IQR, interquatrile range.

### Measures

Participants were asked to complete a questionnaire in self-report format that included questions regarding socio-demographic variables and structured scales to assess activities on the Internet and mobile phone and substance use.

#### Mobile Phone Problem Use Scale (MPPUS-10)

The MPPUS-10 was validated in the present work and was used to assess problematic mobile phone use (18). The MPPUS-10 contains 10 items with a 10-point Likert scale ranging from 1 (“not true at all”) to 10 (“extremely true”). The factor structure of the MPPUS-10 was tested twice in the past. In a study on a group of adolescents (12–17 y.o.), Foersters et al. identified five factors: *Craving, Loss of Control, Withdrawal, Negative Life Consequences*, and *Peer Dependence* (18). Another study, conducted on a sample of adults (18–65 y.o.), resulted in three factors: *Dependence, Withdrawal*, and *Negative Consequences* (37).

#### Mobile Phone Addiction Assessment Questionnaire (MPAAQ, in Polish KBUTK)

The MPAAQ was used to determine the validity of the MPPUS-10. It consisted of 33 items relating to Addiction to Mobile Phone Features, Addiction to Text Messaging and Voice Calls, Need for Acceptance and Closeness, and Indirect Communication. MPAAQ was developed for Polish conditions for youths aged 13–24, showing a reliability coefficient of 0.91. The factor analysis of this scale identified four factors: the need for acceptance and affinity, dependency on mobile phone function, dependency on SMSs and phone calls, and indirect communication (20, 38).

#### Internet Addiction Test (IAT) by Kimberly Young

Internet addiction was assessed using the IAT (39). This questionnaire contains 20 items with a five-point scale ranging from 1 (“very rarely”) to 5 (“very frequently”). We used the Polish version of the IAT (40).

### Statistical analysis

Reliability analysis was performed using Cronbach’s α along with inter-item and corrected item-total correlations (correlation between particular items and total score of all other items).

Confirmatory factor analysis (CFA) was conducted to examine the factor structure of the MPPUS-10 in relation to models of the MPPUS-10 previously postulated in the literature: the five-factor model described by Foerster et al. (18) and the three-factor model described by Nahas et al. (37). The robust maximum likelihood was chosen for the CFA (41, 42). Model fits were compared based on χ2, the Comparative Fit Index (CFI), the Tucker-Lewis Index (TLI), the Standardized Root Mean Residual (SRMR), the Root Mean Dquare Error of Approximation (RMSEA), and the Bayesian Information Criterion (BIC). The BIC was chosen since it was proven to be superior over frequentist fit indices in evaluating the model fit (43). Modification indices were explored for each model.

The validity of the MPPUS-10 in university students was measured as Spearman’s correlation between the MPPUS-10, the MPAAQ (38), and the IAT (39, 40). We included the IAT for the study because previous research has shown that problematic phone use is strongly associated with Internet addiction (19, 44).

The occurrence of mobile phone addiction in the group of students was specified according to the MPAAQ criteria, while problematic mobile phone use was assessed according to the MPPUS-10. The MPPAQ criteria were as follows: mobile phone addiction was specified as MPAAQ score ≥ 70; risk of mobile phone addiction was specified as MPAAQ score > 31 and < 70 (20). MPPUS-10 problematic use was specified as MPPUS-10 score ≥ 59 (37). Based on guidelines provided by Poprawa (40), all participants were divided into two subgroups: a Younger group (< 25 years old) and an Older group (≥ 25 years old). In the Younger group, problematic internet use was specified as IAT score ≥ 80, and the risk of problematic internet use was specified as IAT score ≥ 50 and < 80. In the Older group, problematic internet use was specified as IAT score ≥ 76 and the risk of problematic internet use was specified as IAT score ≥ 42 and < 76. Moreover, the co-occurrence of problematic use of internet and mobile phones was also examined through a correlation analysis of the MPAAQ and IAT scores.

Previous studies have shown that men and women may have different patterns of mobile phone and internet use (45). Therefore, we decided to examine the difference between men and women in the MPPUS-10, MPAAQ, and IAT scores using Mann-Whitney U test or Brunner-Munzel test.

## Results

### Reliability

The MPPUS-10 was reliable, α = 0.78; however, item 10 correlated poorly (r = 0.17) with the rest of the scale. The corrected item-total correlation and MPPUS-10 item correlation matrix are presented in **Table 2**.

**Table 2:** Inter-item and corrected item-total correlations of MPPUS-10 (n=629; α=0.77).

MPPUS-10 ITEM	1	2	3	4	5	6	7	8	9	10	Corrected item-total correlation	α if an item is dropped
1	1										0.40	0.77
2	0.18	1									0.42	0.76
3	0.19	0.33	1								0.31	0.78
4	0.30	0.55	0.32	1							0.59	0.74
5	0.25	0.20	0.16	0.33	1						0.54	0.75
6	0.38	0.23	0.21	0.43	0.44	1					0.59	0.74
7	0.18	0.11	0.03	0.17	0.27	0.32	1				0.34	0.77
8	0.23	0.36	0.25	0.46	0.26	0.39	0.21	1			0.50	0.75
9	0.34	0.18	0.18	0.35	0.73	0.59	0.34	0.37	1		0.63	0.74
10	0.08	0.08	0.00	0.10	0.15	0.08	0.21	0.08	0.17	1	0.17	0.79

Items in MPPUS-10:

1. I have used my mobile phone to make myself feel better when I was feeling down.

2. When out of range for some time, I become preoccupied with the thought of missing a call.

3. If I don’t have a mobile phone, my friends would find it hard to get in touch with me.

4. I feel anxious if I have not checked for messages or switched on my mobile phone for some time.

5. My friends and family complain about my use of the mobile phone.

6. I find myself engaged on the mobile phone for longer periods of time than intended.

7. I am often late for appointments because I’m engaged on the mobile phone when I shouldn’t be.

8. I find it difficult to switch off my mobile phone.

9. I have been told that I spend too much time on my mobile phone.

10. I have received mobile phone bills I could not afford to pay.

### Confirmatory Factor Analysis

**Figure 1:**
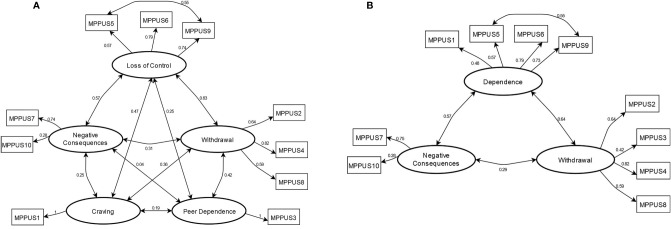
CFA models of **(A)** Foerster’s MPPUS-10 model and **(B)** Nahas’ MPPUS-10 model. Standardized factor loadings are marked with one-sided arrows, while correlations are marked with two-sided arrows. Circles represent latent factors. Models were estimated using Robust Maximum Likelihood.

For both models, free covariance was added between item 5 and item 9, which improved model fit. Fit indices were similar for both models (**Table 3**); nevertheless, the BIC was in favor of Nahas’ model. The factor structures of the models are presented in **Figure 1**.

**Table 3:** Fit indices of the CFA models tested in the study.

	Foerster’s model	Nahas’ model
χ2	64.6	72.6
CFI	0.972	0.970
TLI	0.951	0.956
RMSEA	0.049	0.046
SRMR	0.037	0.040
BIC	26828	26804

### Validity

The correlation between the MPPUS-10 and the MPAAQ was significant (rho = 0.56; p < 0.001), similar to the correlation between the MPPUS-10 and the IAT (rho = 0.54; p < 0.001).

### Occurrence of Technology Addictions

#### Problematic Mobile Phone Use

According to the MPAAQ criteria, 1.1% (n = 6) of all participants were addicted to mobile phones, 50% (n = 264) were in the at-risk group, and 49% (n = 260) did not fulfill criteria for risk or addiction. According to the MPPUS-10 criteria, 9% (n = 47) of all participants were problematic mobile phones users.

#### Problematic Internet Use

According to the IAT criteria, only 0.2% (n = 1) were addicted to internet use, 4.6% (n = 24) were at risk of addiction, and 95.2% (n = 505) did not fulfill criteria for risk or addiction.

There was also significant positive correlation between the IAT and the MPAAQ (rho = 0.38; p < 0.001).

### Between-Gender Differences

#### MPPUS-10

The Mann-Whitney U test showed (U = 35973, p = 0.12) that women (MED = 37; IQR = 20) and men did not differ in terms of MPPUS-10 score (MED = 35; IQR = 20).

#### MPAAQ

The Mann-Whitney U test showed a trend (U = 36322, p = 0.08) showing that women (MED = 32; IQR = 17) scored higher than men (MED = 30; IQR = 17) in the MPAAQ.

#### IAT

The Mann-Whitney U test result for gender and IAT score was not significant (U = 31489, p = 0.29), which shows that men (MED =31; IQR =11) and women (MED = 30; IQR = 9) did not differ in IAT score.

## Discussion

The main objective of this study was to validate the Polish version of the shortened MPPUS-10 (**Table 4**). The validation analysis was conducted on a sample of 530 participants aged 18-38 years. Statistical analyses showed good reliability for the final Polish version (Cronbach’s α = 0.78) and confirmed significant correlation between the MPPUS-10 and the scales previously used in Poland: the MPAAQ (rho = 0.56; p < 0.001) and the IAT (rho = 0.54; p < 0.001).

**Table 4:** Polish version of the 10-item Mobile Phone Problem Use Scale.

Przy każdym stwierdzeniu zaznacz okienko, które najlepiej opisuje, jak poniższe stwierdzenia odnoszą się do Twojej sytuacji, gdzie 1 – “Zdecydowanie nie” a 10 – “Zdecydowanie tak”.
Używałem mojego telefonu komórkowego, żeby poczuć się lepiej, gdy byłem przygnębiony. Kiedy jestem przez jakiś czas poza zasięgiem, martwię się, że mogę przegapić jakiś telefon. Jeśli nie miałbym przy sobie telefonu komórkowego, moim znajomym byłoby trudno się ze mną skontaktować. Odczuwam niepokój, kiedy nie sprawdzam wiadomości lub nie włączam mojego telefonu komórkowego przez pewien czas. Moi znajomi i rodzina narzekają na sposób, w jaki korzystam z telefonu komórkowego. Łapię się na tym, że poświęcam na używanie telefonu komórkowego więcej czasu niż zamierzałem. Często spóźniam się na spotkania, ponieważ zajmuję się telefonem komórkowym wtedy, kiedy nie powinienem. Ciężko mi wyłączyć mój telefon komórkowy. Mówiono mi, że spędzam zbyt dużo czasu używając mojego telefonu komórkowego. Otrzymywałem rachunki za telefon, których nie byłem w stanie opłacić.

The reliability analysis showed that item number 10 of the MPPUS-10 showed low correlation with the remaining items and the total score of the scale (r = 0.17) and that dropping this item would improve the reliability of the questionnaire. In the original version, question 10 was “I have received mobile phone bills I could not afford to pay” and reflected negative life consequences. This item did not conform to Polish culture. A low result for this question was also obtained in studies in Lebanon (37). It should be noted that MPPUS was developed when the amount on bills reflected the intensity of use of the device. Nowadays, a bill does not reflect the frequency of use of the phone. Most users have unlimited access to phone calls, text messages, and the Internet within their subscription. The fact that this question may be out of date was also reported by the respondents during the pilot study. Thus, scientists and clinicians who wish to use the MPPUS-10 could decide to drop item 10 and use the nine-item version.

We obtained acceptable internal consistency, similar to other studies concerning the validity of the MPPUS-10 but lower than those described for the MPPUS-27. A comparison between the Cronbach’s α obtained in our study and those obtained by other researchers is presented in **Table 5**. The difference between our outcome and outcomes described in previous studies may result from cultural differences, statistical population (number, age, education, gender), and number of items.

**Table 5:** Internal consistencies of the MPPUS across different studies.

Cronbach’s α	Number of items	Study group	References
0.93	27	Adults (18 - 85 years)	(16)
0.94	27	Turkish students	(31)
0.97	26	Spanish adolescents (12 – 18 years)	(23)
0.97	26	British adolescents (11-18 years)	(21)
0.90	27	Japan students (18 -25 years)	(29)
0.86	27	German adults (18-46 years)	(26)
0.94	24	Iranian University students	(28)
0.85	10	Swiss adolescents (12-17 years)	(18)
0.94	26	Spanish (16 – 65 years)	(22)
more than 0.7	10	Lebanese adults (18 - 65 years)	(37)
0.78	10	Polish (18-38 years)	presented study

We compared two models of MPPUS-10 using CFA – Foerster’s model and Nahas’ model. In both models, based on the modification indices, free covariance was added between item 5, “My friends and family complain about my use of the mobile phone,” and item 9, “I have been told that I spend too much time on my mobile phone.” These items refer to negative feedback regarding excessive use of the mobile phone, and it is thus not surprising that they were related. Foerster’s model showed better X2, CFI, and SRMR indices, whereas Nahas’ model showed better TLI, RMSEA, and BIC indices. Importantly, participants in studies by Nahas et al. (37) and Foerster et al. (18) differed in terms of age. While Nahas et al. (37) recruited adults 18–65 y.o. (most of whom were 18-34 y.o.), Foerster et al. (18) focused on adolescents aged 12–17 y.o. In the latter study, the authors obtained a factor that they called *Peer Dependence*. Peer dependence may be important during adolescence; however, its significance plausibly decreases with age. Therefore, it might be more important for adolescents than for young adults, who comprise the majority of the sample in our study. In general, the peer dependence factor might be redundant in older samples; however, differences between the fits of the models are small, and researchers in future studies should decide which model better fits their needs. (e.g., whether they need to assess peer dependence or not).

Previous studies of the MPPUS used various criteria to categorize users as problematic vs. non-problematic. For the MPPUS-27, Semtaniuk established three categories: “low to moderate” (27-76 score), “moderate to high” (77-126 score), and “high to severe” (greater than 126) (16, 46). Other authors categorized users according to criteria by Chow et al. (Casual Users, Regular Users, At-Risk Users, and Problematic Users) (47) considering problem users as at-risk users and problematic users (22). Kalhori et al. determined the cut-off point (160) based on psychiatric interview, considered as the “gold standard” (28). However, the authors of the original MPPUS-10 did not find an obvious threshold for differentiating between problematic and non-problematic mobile phone users. According to them, problematic mobile phone use is a continuum, i.e., the higher the score on the MPPUS-10, the more likely mobile phone use is problematic (18). This is reasonable given that (as mentioned in the Introduction) problematic mobile phone use is not a nozological category of a mental disorder and should be evaluated by its intensity rather than using a dichotomic approach.

However, based on a high correlation between the MPPUS-10 and the MPPUS-27 (r=0.95) (18), Nahas et al. extrapolated the cut-off point to a score of 59 to determine problematic smartphone use among Lebanese adults (37). We used the same value in our study. Although we agree with Foerster et al. that problematic mobile use is a continuum of symptom severity rather than a disorder with a recognizable cut-off point, we also do admit that for clinical purposes it is extremely useful to define at least an approximate threshold above which a therapeutic intervention could be recommended. This might be important given well-recognized possible negative mental and somatic consequences of mobile phone overuse. Therefore, we decided to set a cut-off point to provide a proposal for a simple screening tool that is useful for a wide audience in the Polish population. However, we emphasize that the Polish version of the MPPUS-10 is not a diagnostic but a screening tool. Obtaining a score above the proposed threshold should be followed by further, more detailed clinical assessment.

According to the above-mentioned criteria, in the present study, 9% (n = 47) of all participants were problematic users. These results are lower than described using the MPPUS for assessing the prevalence of problematic mobile phone users in other countries, for example, 20.5% among Spanish adults (22), 20.1% among Spanish adolescents, and 20.2% among Lebanese adults (37), but close to the 10% among British adolescents (21). Studies using other criteria than ours identified that 23.4% of Teheran students had mobile phone dependence (MPPUS-24; cut-off point-160) (28) and that, for 25.41% of students of San Francisco State University, there was a “high degree of concern” of problematic mobile phone use (MPPUS-27; scoring greater than 126) (46). However, another Polish study, which was conducted using the *Mobile Phone Problem Use Scale for Adolescents* - MPPUSA (26 items) in a group of adolescents (13–19 years), showed problematic mobile phone use in 6% (48) of the study group. The differences may result from cultural or demographic differences (age, gender, level of education, economic status) and from the questionnaire design (number of questions, accepted criteria). Importantly, participants in our study were students of the Medical University - individuals with a high level of education, including knowledge of the basics of addictions. In addition, members of this group were obliged to broaden the use of new technologies for academic purposes. This could have had a dual impact on our research results. First, all participants were able to correctly understand and interpret the questionnaire’s questions, which can increase the relevance of the result. Secondly, students with knowledge about addictions could consciously or subconsciously suppress or conceal their addiction symptoms. Hypothetically, this could have resulted in the understatement of the scores.

The validity of the Polish version of the MPPUS-10 was assessed by correlation with the *Mobile Phone Addiction Assessment Questionnaire* (MPAAQ; α = 0.95) (20, 38) developed and validated in Poland. We have shown a significant correlation (rho = 0.56; p < 0.001), which confirms the validity of the adopted questionnaire. Despite the high correlation between the raw results of the scales, the diagnosis made on their basis is not unambiguous. Observed differences are probably related to methodological diversity and the criteria used. First of all, each tool is based on different factors (described in the measures section), which are the basis for the diagnostic criteria. Another aspect reflects a significant difference in defining the disorder. It should be noted that the MPPUS was derived to measure problematic mobile phone use, while the MPAAQ was developed to assess mobile phone addiction. Problematic mobile phone use seems to be a broader concept than addiction. Plausibly, participants classified as problematic mobile phone users according to the MPPUS-10 criteria are the whole group of addicted and part of the group at are at risk of addiction according the MPAAQ criteria. In general, the lack of a universal definition of this disorder and differences in diagnostic criteria may cause inconsistencies in data on the prevalence of the phenomenon. Importantly, neither problematic phone use nor mobile phone addiction has been considered as a separate diagnostic category in the international ICD-10, ICD-11, and DSM-5 classifications. There is also no agreement on whether the phenomenon of problematic mobile phone use takes the form of behavioral addiction or only problematic use. In light of our results and based on current literature, it can be concluded that the term “problematic mobile phone use” may be more appropriate than “addiction” (49). Importantly, in the last ten years, the way that telephones are used in Poland has changed significantly. The MPAAQ (used in our study as a comparative scale to confirm the validity of MPPUS-10) contains questions that might have lost their relevance, for example: “I pay very large bills for calls and text messages by mobile phone” or “My mobile phone fees exceed my budget.” This indicates the need to revise current diagnostic tools and perhaps supports the assertion of the authors of the original MPPUS-10 that intensity of mobile use should be treated as a continuous variable.

It should also be noted that the MPPUS was created when smartphones were not yet available. Currently, smartphones have many functions that go beyond talking and writing short text messages. Given that smartphones have many internet-based applications, often with unlimited internet access, it is important to conduct research on problematic mobile phone use in relation to Internet addiction (45, 50, 51). Some researchers have shown that smartphone addiction and Internet addiction overlap (19, 44). In the present study, we have also shown a significant correlation between the MPPUS-10 and the IAT score (rho = 0.54; p < 0.001). This result confirms that problematic mobile phone use is strongly related to problematic Internet use.

In this study, there were no significant differences between men and women in any of the scales used (MPPUS-10, MPPAQ, and IAT). Previous findings of studies on the impact of gender on the problematic use of modern technologies are not conclusive. Some studies have shown that females (Olatz 21, 26, 29, 52) are more at risk of problematic mobile phone use, while other results showed that this risk is higher in males (22, 48). Our results are in line with the studies of Bianchi and Phillips, which showed that both males and females have embraced mobile phones equally (16).

### Limitations

Our study has some important limitations. The study was based on a self-rated questionnaire. Importantly, self-report data regarding the use of modern technologies may result from registered behavior and not actual usage (26). We did not evaluate psychiatric morbidity or stress levels further; therefore, some of the data obtained may be a consequence of these variables rather than directly reflecting the excessive use of mobile phones. Moreover, the study included students at only one university with a relatively high educational level; therefore, the results cannot be generalized to the general population.

Consequently, our results should be interpreted with caution. Further studies are required to confirm our results in the Polish population.

### Conclusion

The main result of this study is the Polish validation and adaptation of the MPPUS-10 scale with confirmed psychometric values. It provides a rapid screening tool to assess the problematic use of smartphones. Due to the fact that the 10^th^ item is outdated in Polish culture and correlates poorly with the other items, we suggest the use of a nine-item version of the MPPUS with a cut-off score extrapolated for this number of items: ≥53 points. We confirmed that problematic mobile phone use is strongly related to problematic Internet use. Furthermore, problematic use of modern technologies is not associated with gender. The present study also indicates the need for revision of available diagnostic tools and for further studies.

## Data Availability Statement

The raw data supporting the conclusions of this article will be made available by the authors, without undue reservation, to any qualified researcher.

## Ethics Statement

The studies involving human participants were reviewed and approved by Ethics Committee of Medical University of Warsaw. Written informed consent for participation was not required for this study in accordance with the national legislation and the institutional requirements.

## Author Contributions

All authors contributed to the conceptualization and design of the analyses. AM, AKl, AJ, MA, and MW designed the study and wrote the protocol. AM, MD-J, AKl, AJ, MA, AKu, and PS contributed to the data collection. JS, AM, AKl, AJ, and MW took responsibility for conducting analyses. AM, MD-J, AKl, AJ, MA, and MW managed the literature search. AM and JS wrote the first draft of the manuscript. AM, MD-J, AKl, AJ, MA, AKu, PS, and MW provided substantive and conceptual feedback on all drafts. All authors contributed to and have approved the final manuscript.

## Funding

This research was funded by a National Bureau for Drug Prevention grant (194/HM/2017, 166/HBK/2018).

## Conflict of Interest

The authors declare that the research was conducted in the absence of any commercial or financial relationships that could be construed as a potential conflict of interest.
